# Refuting the sensational claim of a Hopewell-ending cosmic airburst

**DOI:** 10.1038/s41598-023-39866-0

**Published:** 2023-08-09

**Authors:** Kevin C. Nolan, Andrew Weiland, Bradley T. Lepper, Jennifer Aultman, Laura R. Murphy, Bret J. Ruby, Kevin Schwarz, Matthew Davidson, DeeAnne Wymer, Timothy D. Everhart, Anthony M. Krus, Timothy J. McCoy

**Affiliations:** 1 Applied Anthropology Laboratories, College of Sciences and Humanities, Ball State University, Muncie, USA; 2 Midwest Archaeological Center, National Park Service, Lincoln, USA; 3 World Heritage Program, Ohio History Connection, Columbus, USA; 4 Department of Sociology and Anthropology, Washburn University, Topeka, USA; 5 Hopewell Culture National Historic Park, National Park Service, Chillicothe, USA; 6 ASC Group, Inc., Columbus, USA; 7 Department of Anthropology, University of Kentucky, Lexington, USA; 8 Susquehanna River Archaeological Center, Waverly, USA; 9 Department of Anthropology and Sociology, University of South Dakota, Vermillion, USA; 10 Curator of Meteorites, Smithsonian National Museum of Natural History, Washington, USA

**Keywords:** Environmental impact, Natural hazards, Meteoritics

## Introduction

**arising from**: K. B. Tankersley et al.; *Scientific Reports* 10.1038/s41598-022-05758-y (2022).

Tankersley et al.^1^ claim a cosmic airburst over modern-day Cincinnati, Ohio in the 3rd or fourth century CE catalyzed the decline of Hopewell culture. This claim is extraordinary in the face of hundreds of archaeological investigations in the Middle Ohio River Valley (MORV) that have heretofore provided no evidence of a widespread cataclysm or “social decline” in need of explanation. Tankersley et al. misrepresent primary sources, conflate discrete archaeological contexts, improperly use chronological analyses, insufficiently describe methods, and inaccurately characterize the source of supposed extraterrestrial materials to support an incorrect conclusion. While charcoal and burned soils are found on virtually all excavated Middle Woodland archaeological sites in the region, these have prosaic explanations. Many of the burned “habitation surfaces” mentioned are actually prepared surfaces for ceremonial fires, not the result of a synchronous regional catastrophe. Radiocarbon dated samples from one context are mistakenly attributed to distinct and unrelated contexts. The chronological analysis does not support the notion of a single event spanning 15,000 km^2^. The composition of their supposed extraterrestrial materials is inconsistent with an origin in comet or asteroid events. In sum, there is no evidence to support the conclusion that a comet exploded over modern-day Cincinnati in the third or fourth century CE.

Attempts to associate extraterrestrial events as the direct cause of various ancient cultural declines, i.e. “cosmic catastrophism,” have appeared in several recent papers^2,3^. These catastrophist narratives have met consistent challenge on evidentiary, methodological, and theoretical grounds^4,5^. These scenarios oversimplify complex and dynamic human–environment interactions and are steeped in pseudoscientific beliefs rather than anthropological theories of social decline that can be tested using the archaeological record. Tankersley et al.’s proposition that a comet or meteor airburst caused the decline of the Hopewell culture is the latest example of a cosmic claim dooming a culture with no substantive archaeological or geologic evidence. Instead, the Hopewell archaeological record demonstrates continued habitation with gradual sociopolitical and economic reorganization (e.g., a cessation in large ceremonial earthwork construction) and changes in settlement patterns within the MORV^6–11^.

Tankersley and colleagues’ argument depends on several misinterpretations and mischaracterizations of the Hopewell archaeological record to arrive at their conclusion. There are no catastrophically burned, fire-hardened, charcoal-rich habitation surfaces documented at any Hopewell site. The soil profiles published in the body of the article (Figs. 2–12 in ^1^) and the soil descriptions in their supplemental material (Tables S5, S7, S10, S13, S16, S18, S21, S23, S25, S28, and S30) do not show evidence of in situ burning as claimed. The burned surfaces referenced in the article are in fact ceremonial basins, localized burned areas, or burned floors within mounds^12,13^. In aggregate, these burned areas do not support widespread regional burning by a catastrophic event, but a series of intentionally burned surfaces as a regionally-distributed socioreligious practice.

## Archaeological context

Tankersley et al.^1^ make numerous errors concerning archaeological data and context that undermine the veracity of their claims; a few of these are highlighted herein. First, they conflate the stratigraphy and results of two discrete test units (Units A and B) from Tankersley’s own work at the Jennison-Guard site (12D246), Lawrenceburg, Indiana (Table 1). The primary technical reports for this fieldwork^14,15^ indicate that the unit shown in Fig. 4, Unit B, was a 1 m × 1 m unit excavated in 2019. Tankersley’s final report^14^ (p. 7) on the excavations notes “No artifacts were recovered from the 2019 1-m^2^ unit.” The artifacts are reported as coming from the “2020 1 × 2 m^2^ [sic]” Unit A in Table ﻿1 of the final report^14^. This list is identical to the artifact inventory in the preliminary report’s^15^ appendix without provenience information. Thus, all of the artifacts recovered were from Unit A, while the claimed evidence for “piles of carbonized timbers” (see Fig. 4 of ref. ^1^) and all the Pt and Ir samples reported were recovered from the culturally sterile Unit B, approximately 10 m away on a dynamic floodplain. Second, the depth of Unit B is represented variously as 1.6 m and 2 m, while the changes in soil horizons are inconsistently presented. The layer (1.57–1.6 m) that produced the charcoal-impregnated clay, which they believe represents burned house wall timbers, is characterized as being “Pre-habitation” (Table S9) with the same date range as the layer above (Table S10). This 0.38 m to 1.57 m series of horizons (C1, C2, and C3) are presented as dating to 1–400 CE (Table S9) and 259–410 CE (Table S10). Thus, there is no substantiated connection between the proposed proxies for the cosmic airburst (burned timbers and elevated Pt and Ir in Unit B) and the Hopewell artifacts recovered from Unit A; it is not proper archaeological practice to copy and paste an artifact assemblage into another context. Thus, no evidence presented in the article^1^ or the technical reports^14,15^ supports catastrophic burning at Jennison-Guard, let alone a synchronous regional-scale catastrophe.

**Table 1:**
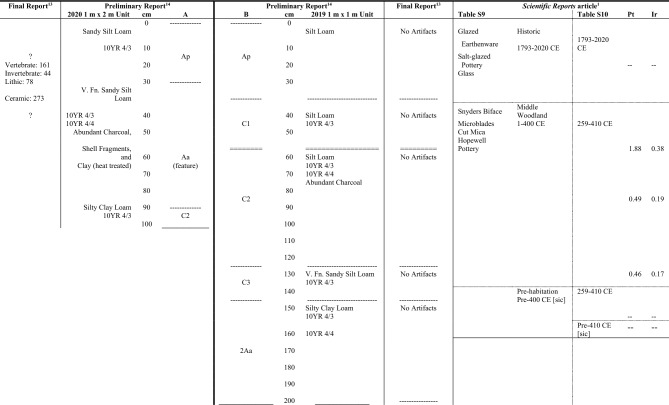
A comparison of presentation of evidence from the Jennison–Guard site by Tankersley et al.^1^, Tankersley^13^, and Tankersley et al.^14^

Horizontal lines are representations of horizon breaks in various units and the diverse and problematic ways the data are presented for each in the cited reports and article.

**Figure 1:**
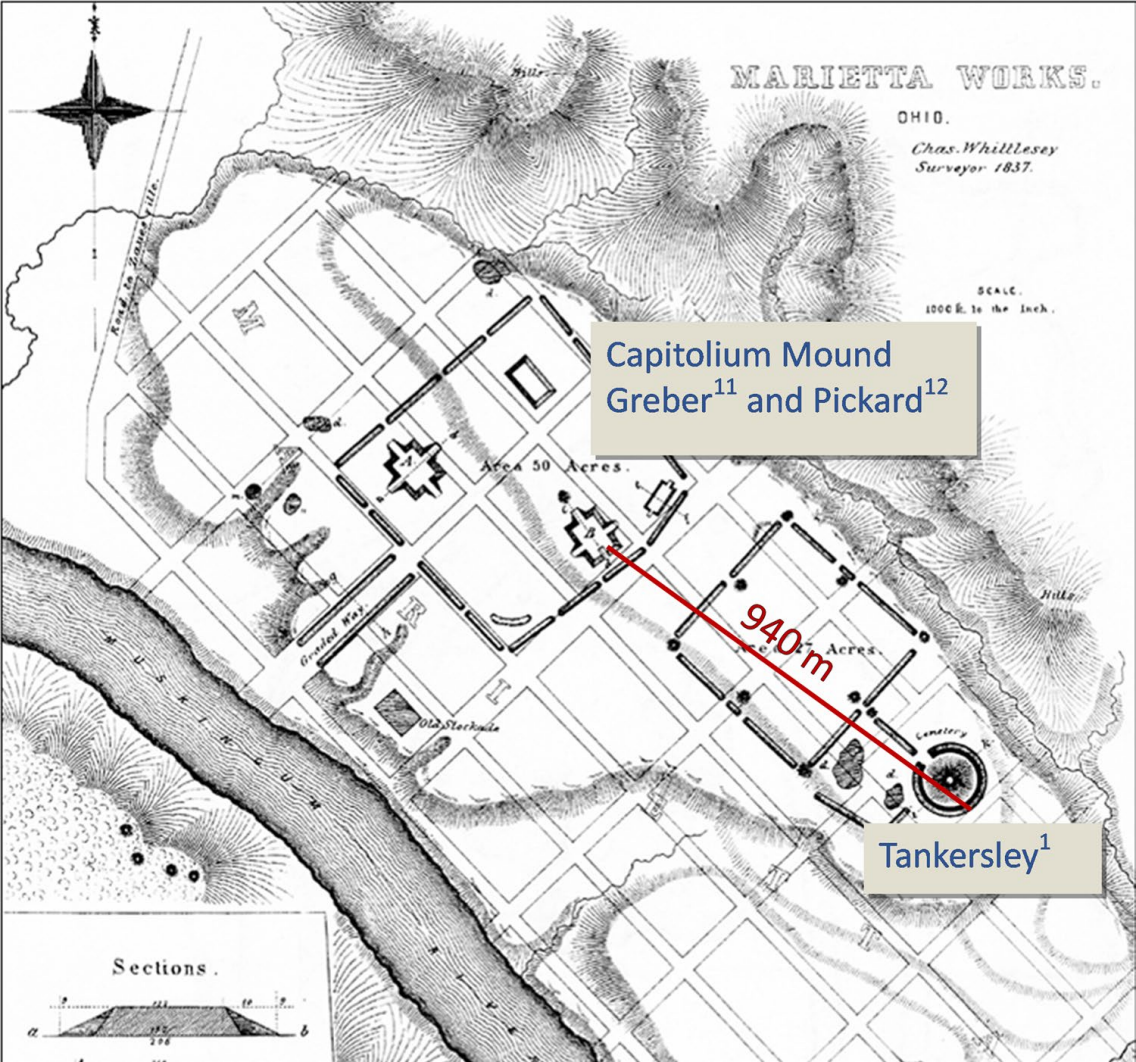
Comparison of the Location of the fire-hardened floor investigated by Greber^11^ and Pickard^12^ within Capitolium Mound and the location of the coordinates from Tankersley et al.’s Table S1^1^ for their Marietta sample over Squier and Davis^15^ map of the site.

Another example of conflated archaeological context is from the Marietta earthworks site, Marietta, Ohio, where Tankersley et al.^1^ report (Table S1) they sampled from the area of the large circular enclosure (Mound A) at the southeast end of the earthworks complex, approximately 940 m from the fire-hardened floor referenced as evidence of catastrophic burning (Fig. 1). Their elemental analysis and identification of pallasites and presumed microspherules of cosmic origin is in an artificially constructed earthwork (presumably within the mound or embankment at the coordinates listed in Table S1, but this is not specified) nearly a kilometer away from the other supposed evidence of the catastrophic event. Tankersley et al. do not report new radiocarbon dates for the location they actually sampled, but instead appropriate the radiocarbon dates from Greber^12^ and Pickard^13^, which are from a completely different element of the Marietta monumental landscape. These radiocarbon dates were produced from organic material derived from four separate features in the Hopewellian platform mound best known as the Capitolium Mound. There is no established relationship between the construction of the intentionally burned clay platform within Capitolium Mound and their samples from an unspecified context within Mound A. Minimally, both sets of deposits (like all the cultural deposits analyzed by Tankersley et al.^1^) date broadly to the Middle Woodland period. However, the origin and formation of each earthwork and the strata within each cannot be assumed to be synchronous. In fact, the probability of synchronous construction of both monumental structures, to include synchroneity between the Ir/Pt anomaly layer of Mound A and the prepared ceremonially fired surface within Capitolium Mound, is quite low given the magnitude of the constructions, the scope of the Marietta monumental landscape, and the wide probability distribution for the Capitolium dates alone (see Fig. 21 in ^1^).

Tankersley et al.^1^ make no attempt to establish this chronological relationship, and provide no justification for their reinterpretation of the prepared surfaces, here and elsewhere, as “habitation surfaces.” With the absence of evidence, the only thing that ties these discrete contexts together is that the conclusion of the authors requires this relationship. Given the lack of relationship, there is no support for an event that took place over seconds (the airburst) to days or weeks (the fires). Space precludes enumeration of the similar errors present for most of the sites discussed in the *Scientific Reports* article.

Additional discrepancies occur between Tankersley et al.’s text descriptions, figures, and supplemental material. Specifically, none of the figures of soil profiles bear any sign of in situ, fire-hardened habitation surfaces, and in most cases, labels of “Charcoal” are imposed onto profiles with no identifiable carbonized material present in the profile. Moreover, after careful review of the primary reports of their investigations (e.g., Fig. 20, Fig. 23, Fig. 24 in ^14^), the authors fail to show an association of burned habitation surfaces with Ir/Pt anomalies and microspherules and that the alleged “remains of burned Hopewell structures” were “swept into piles of carbonized timbers and thatch, fire-hardened daub, and thermally damaged artifacts” (p. 10 in ^1^; see ^17^). In the absence of such evidence, the claim of a catastrophic event recorded in the soil profiles is not credible.

## Chronological modeling

Tankersley et al.’s chronological modeling is insufficiently explained (methods, model code, etc.), incorrectly characterized, and does not support the inference of a single event. While characterized as “Bayesian adjustment” (see Fig. 21, Figure S2, Figure S5, Figure S7, Figure S11, Figure S15 in ^1^), no Bayesian models are included in the article or supplemental material. No OxCal code is included, and the functions used to generate their graphs are not revealed. (K.C.N. requested these details from Tankersley, who declined to share these details prior to submission.) Based on the type of OxCal graphs shown, it appears the authors calculated a weighted mean of the radiocarbon dates from the presumed airburst strata across four different sites. Despite the characterization in the article, weighted means is not one of the Bayesian functions in OxCal. This method only makes sense to date a target event (e.g., an airburst) if the dated samples died at the same time as the event. The 20 radiocarbon measurements that Tankersley et al. claim directly date their airburst event cannot feasibly be interpreted as the same age. This sample of dates fails to pass a *Χ*^2^ test^18^ (T = 42.899; df-19; T’(0.05) = 30.1) indicating that the dated samples were deposited over a prolonged period of time. Therefore, the dated samples from these strata cannot be presumed to date a single event and cannot support the airburst hypothesis. To the contrary, the chronological analysis demonstrates that their multi-proxy evidence of the airburst accumulated via many discrete events over decades to centuries.

## Cosmic geochemistry

**Figure 2:**
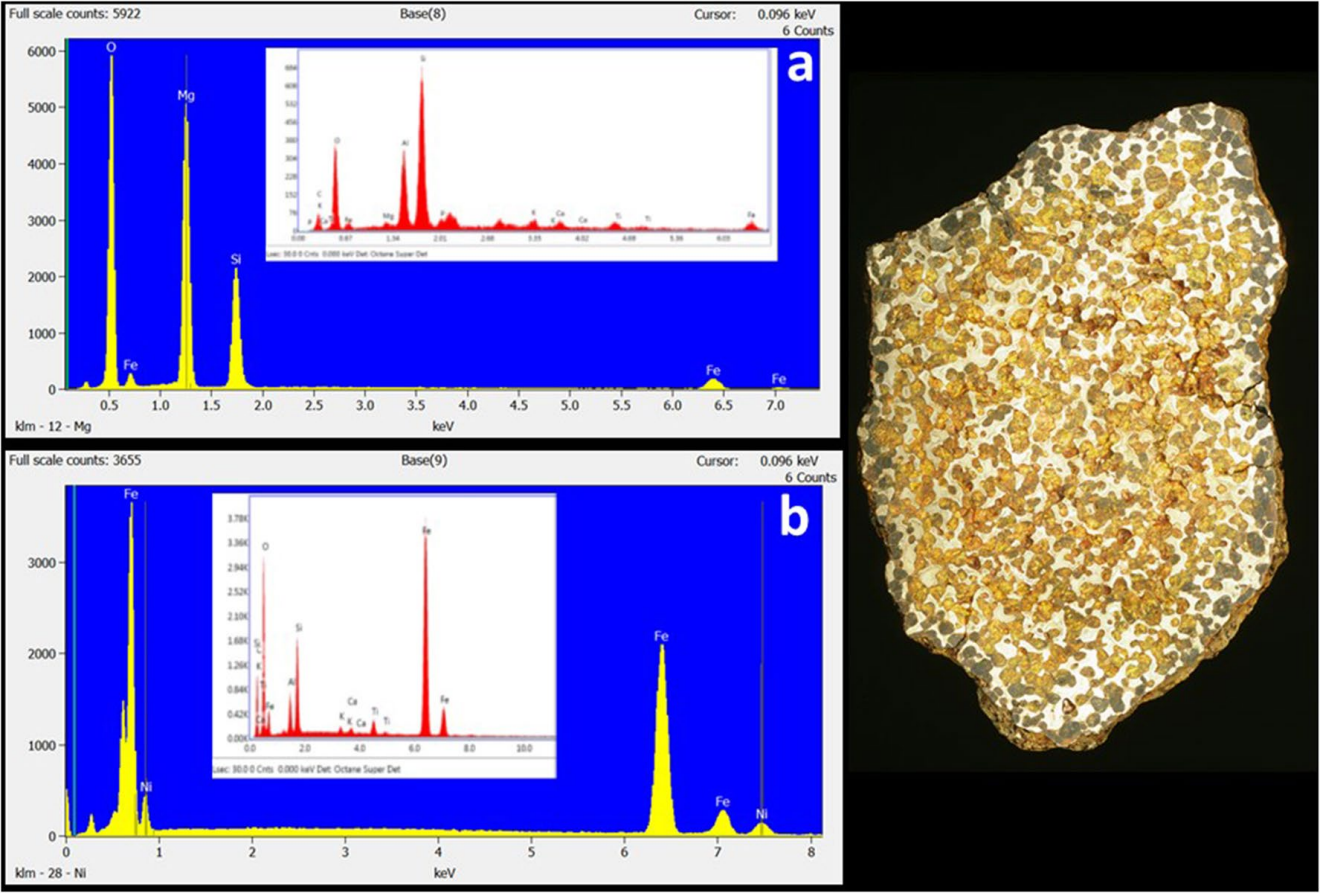
Comparison of EDS spectra for Fe-rich (metal) and Si-rich (silicate) spectra from the Brenham pallasite (pictured right) in the collections of the Smithsonian Institution collected with a FEI Nova NanoSEM 600 at the Smithsonian with spectra of Tankersley et al.’s Fig. 15.^1^ Brenham consists of olivine ((Mg,Fe)_2_SiO_4_) and Fe,Ni metal. EDS spectra of olivine (**a**) contains prominent peaks for Si, Mg, O and Fe, whereas those of Tankersley et al. (a inset) contain minimal Mg and abundant Al, with lesser K, Ca, and Ti, likely indicative of a local soil composition. EDS spectra of Fe,Ni metal (**b**) exhibits a significant Ni peak, which is weak or absent in the spectra (**b** inset) of Tankersley et al., which contains abundant O, Si, Al, K, Ca and Ti, suggesting an iron oxide composition with a local soil component.

Tankersley et al.^1^ misinterpret recent evidence for high-temperature components in comets leading to problematic geochemical analyses and interpretations of supposed pallasite samples. While samples of comet 81P/Wild2 collected by the Stardust mission contain high-temperature components formed in the solar nebula prior to planetary accretion, melting and differentiation^19^, materials formed as a result of planetary differentiation, such as pallasites, are unknown from cometary samples. Comets likely never reached temperatures much above the freezing point of water^20^, whereas the formation of pallasites required extensive melting of asteroids at temperatures ≥ 1300°C^21^. Moreover, isotopic signatures separate meteorites into two groups, thought to form in the inner and outer Solar System and isolated by the formation of Jupiter^22,23^. These isotopic signatures indicate that pallasites formed in the inner Solar System, isolated from the outer Solar System and the comet-forming region. Main group pallasites, like the Brenham meteorite argued to have been used by the Hopewell, are mixtures of olivine ((Mg,Fe)_2_SiO_4_) and Fe,Ni metal (Fig. 2)^24^. In contrast, the Si-rich and Fe-rich spheres illustrated by Tankersley et al. (Fig. 15)^1^ are depleted in or lack Mg and Ni, respectively. This suggests that the Si-rich and Fe-rich spheres are not derived from pallasites, and more likely represent local soil chemistry. Thus, Tankersley et al.^1^ mischaracterize the composition and life histories of comets, and they misidentify pallasites. Had Tankersley et al.^1^ correctly identified pallasites, it would in fact be evidence against a comet airburst. Neuhäuser and Neuhäuser^25^ using different sources also challenged Tankersley et al.’s identification of a comet, and Tankersley et al.^26^ acknowledge that the airburst may have been an asteroid. Our analysis demonstrates even this concession relies on morphological identification of spherules as cosmic in origin, without associated and necessary chemical or isotopic evidence of detritus from the explosion of an extraterrestrial body, neither comet nor asteroid, in the data Tankersley et al. present.

While not a comprehensive review of all of the issues with Tankersley et al.’s “Hopewell airburst event,” this brief summary demonstrates the systematic flaws in the analysis and interpretation of archaeological data, chronological data, and cosmic geochemistry. We find that their presentation and argument:

1. Does not support claims of a catastrophic regional burning,
2. Does not demonstrate their evidence is, in fact, synchronous,
3. Does not demonstrate that microspherules are related to meteorites,
4. Mistakenly claims that pallasite fragments could have originated in comets, and
5. Does not provide evidence for a widespread decline in Hopewell culture.

In short, their observations fail to demonstrate any aspect of this cosmic catastrophe.

## Data Availability

The data used in this contribution are the original data presented in Tankersley et al.’s paper and supplemental material, the original technical reports required to be submitted by Tankersley to the Indiana Division of Historic Preservation and Archaeology (DHPA), and other references cited herein. All reports and files available upon request to the corresponding author, or through the DHPA, and the original publication.
